# Needs and wishes of Gen Z medical students: a prospective cross sectional single center study

**DOI:** 10.1007/s00423-026-04024-3

**Published:** 2026-03-27

**Authors:** S. Mohr, E Khajeh, R Nikbakhsh, J. Fellhofer-Hofer, N Halama, F. Nickel, A. Mehrabi, Fee Klupp

**Affiliations:** 1 Medical Faculty, Department of Anesthesiology, Heidelberg University, University Hospital Heidelberg, Im Neuenheimer Feld 420, Heidelberg, 69120 Germany; 2 Department of General, Medical Faculty, Visceral and Transplantation Surgery, Heidelberg University, University Hospital Heidelberg, Im Neuenheimer Feld 420, Heidelberg, 69120 Germany; 3 Medical Oncology, National Center for Tumor Diseases (NCT), Institute of Immunology, University Hospital Heidelberg, Im Neuenheimer Feld 460, Heidelberg, 69120 Germany; 4 Department of Translational Immunotherapy, German Cancer Research Center (DKFZ), Im Neuenheimer Feld 280, Heidelberg, 69120 Germany; 5 Department of General, Visceral and Thoracic Surgery, University Medical Center Hamburg – Eppendorf, Martinistr. 52, Hamburg, 20246 Germany

**Keywords:** Medical education, Undergraduate, Generation Z, Gen Z, Future work, Study conditions, Working conditions, Career ambitions, Future physicians

## Abstract

**Background:**

Generation Z (Gen Z) is now entering the labor market with new expectations from their employers. In the healthcare sector, particularly in hospitals and medical practices, employers must adapt to the needs of this young generation to succeed. We assessed the wishes and expectations of Gen Z employees in medicine with regard to their motivational status, learning behavior, work-life balance, and career plans. We also considered the challenges these expectations present to employers.

**Methods:**

Fourth-year medical students were recruited for participation in this study. Study participation was voluntary and anonymous. Participants were questioned on their expectations using an evaluation questionnaire with either dichotomous or multiple-response options. The participants’ answers were analyzed using a five-point Likert scale.

**Results:**

A total of 137 fourth-year medical students participated in this study. The mean participant age was 23 years. The most common reasons for studying medicine were a desire to help others (69%) and having a secure job later on (63%). The participants also favored online learning tools over medical books. Regarding the preferred specialty after their examination, 39% of students wanted to go into surgery and 23% wanted to go into anesthesia. Participants considered the most important work attributes to be satisfaction (96%) and fulfillment (94%), while having power (11%) and doing research (24%) were considered the least important.

**Conclusions:**

This study reveals how Gen Z medical students have a different mindset to their work than students from earlier generations did, with distinct visions of their professional future. As a result, employers and medical educators face new expectations and demands from their staff and students. To meet the needs of Gen Z students, medical teaching methods must be rethought and adapted as necessary.

## Introduction

### The generation concept

‘Generation’ refers to a specific age group that is characterized by the historical or cultural events of their childhood or adolescence. These events include wars, emergencies (such as the post-war period), and political upheavals (such as the fall of communism). Social trends, such as the 1968 movement or digitalization, also influence the everyday lives of the generation that grow up with these changes. Significant differences have been observed between different generations, and these differences manifest as different objectives, different interactions with each other, and a different value base [1–3]. These generational differences are more heuristic than deterministic.

### Generation Z

Everyone born between the mid-nineties and 2012 belongs to Generation (Gen) Z, which means our current medical students and postgraduate physicians belong to this generation. Gen Z are generally thought of as digital natives and are seen to be creative and sustainable as well as assuming social responsibility and striving for safety [4]. For these individuals, separating work and leisure is important, and they focus on personal development, which can have a negative impact on the employment market, especially in the medical sector. These individuals may also exhibit different learning behaviors to earlier generations because of digitalization [5].

### Generations X and Y

Individuals belonging to Gen Y (millennials) were born between 1980 and 1995, and were shaped by the internet boom, digital transformation, and economic globalization. They are seen to have a strong need for flexibility and belonging [6–8]. Individuals belonging to Gen X were born between 1965 and 1979, and are seen to be savvier with technology, more independent, and less loyal to the institution, while seeking balance between work and lifestyle [9–11]. Current medical teachers, supervisors, and chiefs of Gen Z belong to Gen Y and Gen X. Because of these generational differences, behavior-based conflicts, value-based conflicts, and identity-based conflicts can arise [12].

### Current state of literature and research questions

Gen Z now comprises the majority of undergraduate medical students and early postgraduate trainees [13]. Several authors have argued that the formative experiences of Gen Z—which include pervasive digital technology, economic insecurity, climate anxiety, social media, and school closures due to COVID-19—have shaped the distinctive work and learning expectations of this generation. Based on these experiences, a stronger emphasis has been placed on mental health and well-being [13], learning with variable digital tools [14], meaningful work, and work-life balance [15]. These generational tendencies do not replace individual or cultural variation, but they provide a useful lens for interpreting current medical education data.

The needs and expectations of Gen Z nursing students has been investigated. Whether Gen Z students choose to pursue a career in nursing is influenced by a combination of economic motives (job security, advancement, financial independence) and idealistic-vocational motives (helping others, meaningful work, empathy, hero image) [16]. But there is a clear gap between expectations and reality. Students are aware of the burdens of a nursing career, but the concrete precariousness (wages, hours, compatibility with private life) and the possible consequences for their own lives. Shorey et al. assessed different health care students in their scoping review, but focusing mainly on nursing students. The expectations of health care students have been assessed in a scoping review, but focus has mainly been on nursing students [17–19] and the needs and wishes of Gen Z medical students are not well described. To address this gap, we defined two superordinate domains with five exploratory research questions to investigate the motivations, learning behaviors, and career expectations of fourth-year Gen Z medical students.

The following five exploratory research questions were investigated, grouped into two domains:

Domain 1: study assessment

1. Motivational status: What intrinsic and extrinsic motives influence the decision of Gen Z students in Germany to begin studying medicine?
2. Learning culture: What kind of learning culture do medical students have, particularly with regard to learning materials, learning locations, and learning times?

Domain 2: future workplace assessment

3. Subject-specific interests and experiences: Which medical discipline is favored, and what experience has already been gained in this field?
4. Career ambitions: What are the professional and academic career ambitions of medical students in their fourth year of study?
5. Value concepts and wishes for the future workplace: What values and wishes do medical students have for their future workplace?

## Materials and methods

### Participant recruitment

The study was designed as an exploratory cross-sectional survey. Participants were recruited from 1 September 2024 to 30 November 2024 from the pool of students enrolled in their fourth year of medical studies at Heidelberg Medical School, and no exclusions were made based on cultural background. We only included medical students because students from other degree programs would not have the same future working conditions (for example, office work can be done from home whereas hospital work requires on-site presence). Study participation was voluntary and blinded, and written informed consent was obtained from every participant. Specifically, all fourth-year medical students in the surgical semester were contacted by email and invited to participate in the study. A consent form was attached to the email. Those agreeing to participate signed the consent form and returned it by email before completing the questionnaire. Questionnaires were only sent to those students who returned their consent form, and there were no disadvantages for students who chose not to participate. Each student completed the questionnaire once. The study was approved by local ethics committee (S-465/2024).

### Questionnaire

A self-designed questionnaire was used to question the participants about their motivational status and career plans (Supplement Fig. 1). The questions were composed by several medical teachers from different departments (surgery, anesthesia, radiology, oncology) together with two student representatives. The questionnaire comprised 30 items including 17 dichotomous (yes/no) questions, four multiple-choice questions, seven open questions, and two 5-point Likert-scale questions (1 = strongly agree to 5 = strongly disagree) each with 21 items. Content validity was ensured through expert review and pilot testing by 10 medical students. Internal consistency measures such as Cronbach’s alpha were not applicable because the items were heterogeneous and there were no multi-item scales.

### Statistical analysis

Differences across multiple categorical and ordinal variables in the questionnaire responses were analyzed using various statistical tests. The Chi-square test and Fisher’s exact test were used to evaluate the association between categorical responses, particularly when the expected frequencies were low, to ensure robust statistical conclusions. Ordinal data, such as Likert-scale responses, were analyzed using the Wilcoxon rank-sum test to compare distributions between groups. For descriptive comparisons across survey domains, Likert-scale responses were additionally grouped into disagreement (scores 1–3) and agreement (scores 4–5) categories to facilitate interpretation of overall response tendencies. Normally distributed continuous variables were assessed using Student’s t-tests to compare mean differences. Because of the exploratory nature of the study, no adjustment for multiple testing was applied. A p-value of < 0.05 was considered statistically significant. Statistical analyses were conducted using RStudio and graphs were created using GraphPad Prism (version 9).

## Results

In total, 137 fourth-year medical students participated in this study. The mean age of these participants was 23 years, and 40% (*n* = 55) were male, 60% (*n* = 83) were female, and none had a diverse gender identity. The participants answered five questions from two domains: study assessment (domain 1) and future workplace assessment (domain 2). The following analyses summarize descriptive trends in students’ responses across the surveyed domains:

### Domain 1: study assessment

1. Motivational status: What intrinsic and extrinsic motives influence Gen Z students in Germany to begin studying medicine?

**Fig. 1:**
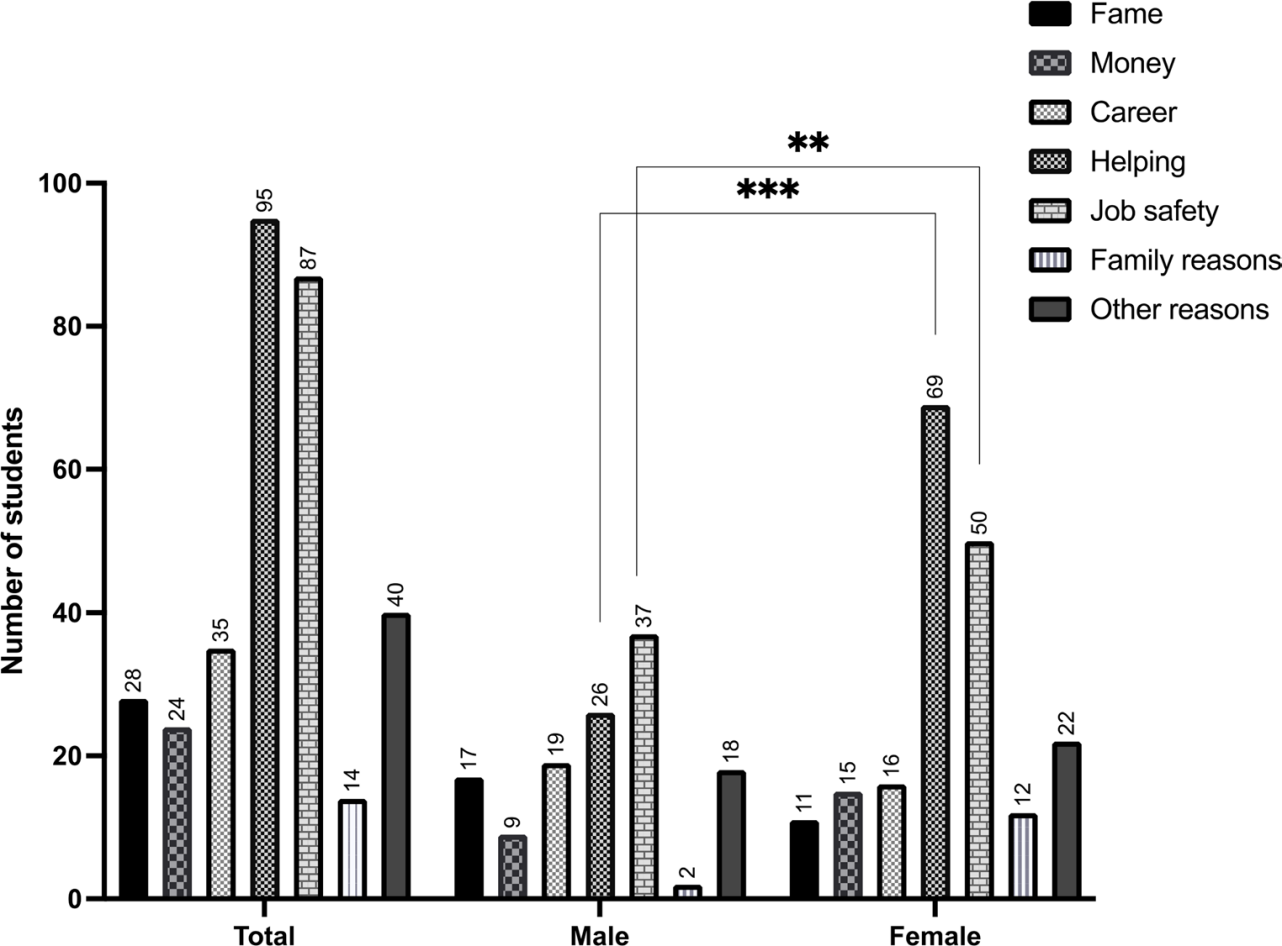
Responses to the question “Why did you decide to study medicine back then?” ** P < 0.01, *** P < 0.001

The participants were allowed to give multiple responses to this question. Responses to the question “Why did you decide to study medicine back then?” (Fig. 1) showed that 69% (*n* = 95) of participants wanted to help people. This answer was chosen significantly more often in woman (*n* = 69) compared to male students (*n* = 26) (*p* = 0.001). The next most popular reason for studying medicine was job safety (63%, *n* = 87), followed by making a career (25%, *n* = 35), fame and respect (20%, *n* = 28), money (17%, *n* = 24), and family reasons such as parents or other family members being physicians (10%, *n* = 14). Female students reported job security as a motivation more frequently than male students (*p* = 0.01). In addition, 29% (*n* = 40) of participants listed other reasons for studying medicine, including professional interest, scientific interest, medicine being their dream job, and medicine providing diverse opportunities.

2. Learning culture: What kind of learning culture do medical students have, particularly with regard to learning materials, learning locations, and learning times?

**Fig. 2:**
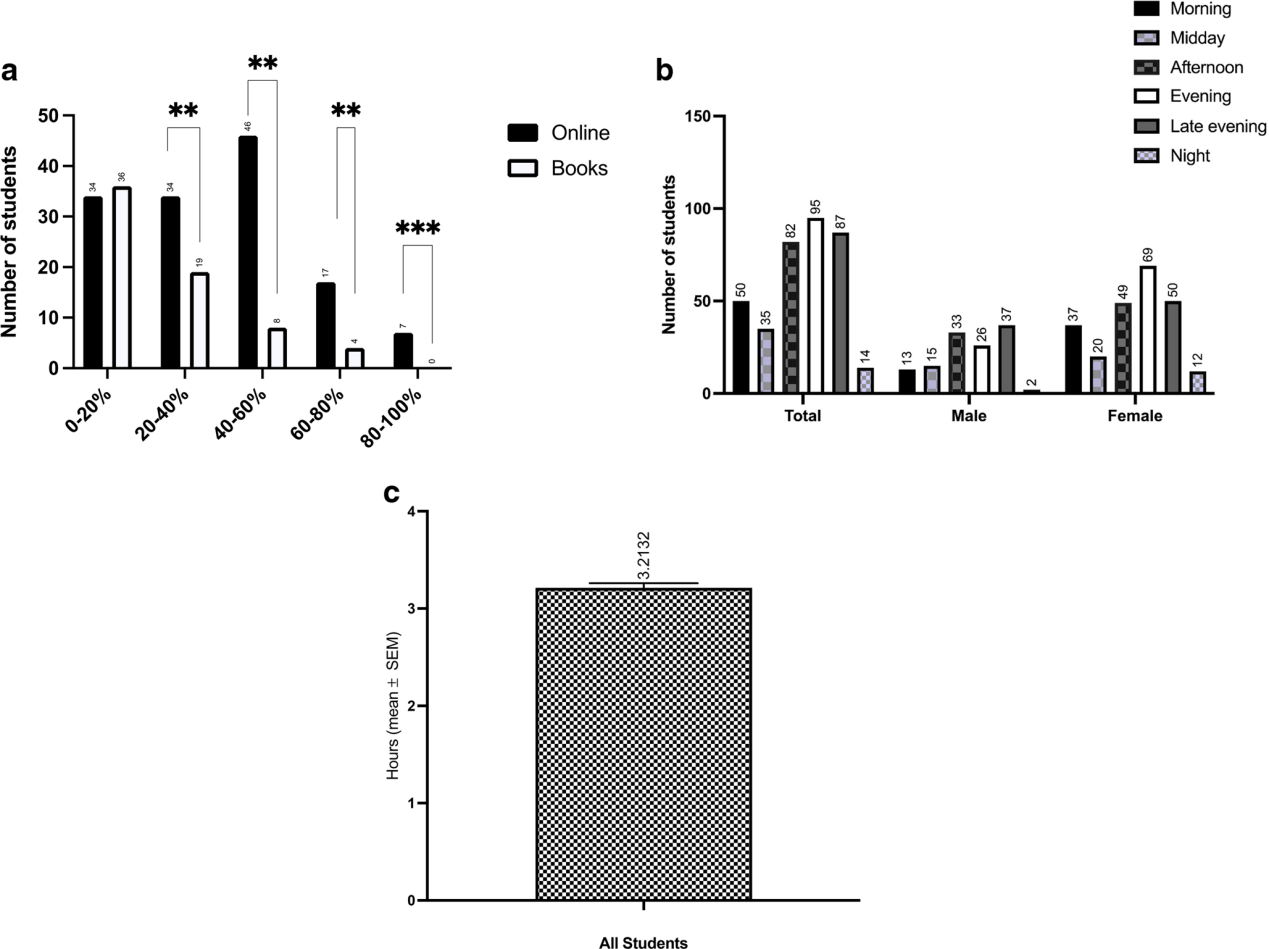
**a** Responses to the question “From which sources do you learn and to what extent?” Possible answers were 0–20%, 20–40%, 40–60%, 60–80%, and 80–100%. ** P < 0.01, *** P < 0.001. **b **Responses to the question “At what times do you mainly study?” Possible answers were in the morning (6 a.m. to 12 p.m.), midday (12–2 p.m.), afternoon (2–6 p.m.), evening (6–9 p.m.), late evening (10 p.m. to 12 a.m.), and night (12–3 a.m.). **c** Responses to the question “After how many hours of studying in a row do you need a break?”

The next questions assessed learning behaviors: “From which sources do you learn?” (Figure 2a). Online learning offers are the predominant chosen learning tools (51%; n=70). Using medical books accounts only for a small proportion the dominant source (8,8%; n = 12). 51% (n=70) prefer online media to 40-100% as a source. Moreover, it was asked, if the students want to increase online learning lessons. 34% (n=45) of the students stated that extending asynchronous online learning sessions were useful for them but 65% (n=86), stated that they don´t want more online lessons. 9 % (n=12) answered that 50-100 % of the studies should be performed online so that they can stay at home. Moreover, they confirmed positively that a feedback culture is very meaningful to them (94%; n=123). 

The students were also asked if they wanted more lessons to be given online; 34% (*n* = 45) stated that more asynchronous online learning sessions would be useful for them, whereas 65% (*n* = 86) stated that more online lessons would not help. Only 9% (*n* = 12) of participants said that 50–100% of studies should be performed online so that they could stay at home. Most students reported that feedback during learning was important to them (94%, *n* = 123).

Most students preferred to learn in the evening. Six to 9 p.m. in the evening was preferred by most students (69%, *n* = 95), followed by the late evening (10 p.m. to 12 a.m.; 63%, *n* = 87), the afternoon (2–6 p.m.; 60%, *n* = 82), the morning (6 a.m. to 12 p.m.; 36%, *n* = 50), midday (12–3 p.m.; 25%, *n* = 35), and finally late night (12 a.m. to 3 a.m.; 10%, *n* = 14). The students were allowed to give more than one answer for this question (Fig. 2b). The mean number of hours the students said they could study before needing a break was 3.2 h (Fig. 2c).

### Domain 2: future workplace assessment

3. Subject-specific interests and experiences: Which medical discipline is favored, and what experience has already been gained in this field?

**Fig. 3:**
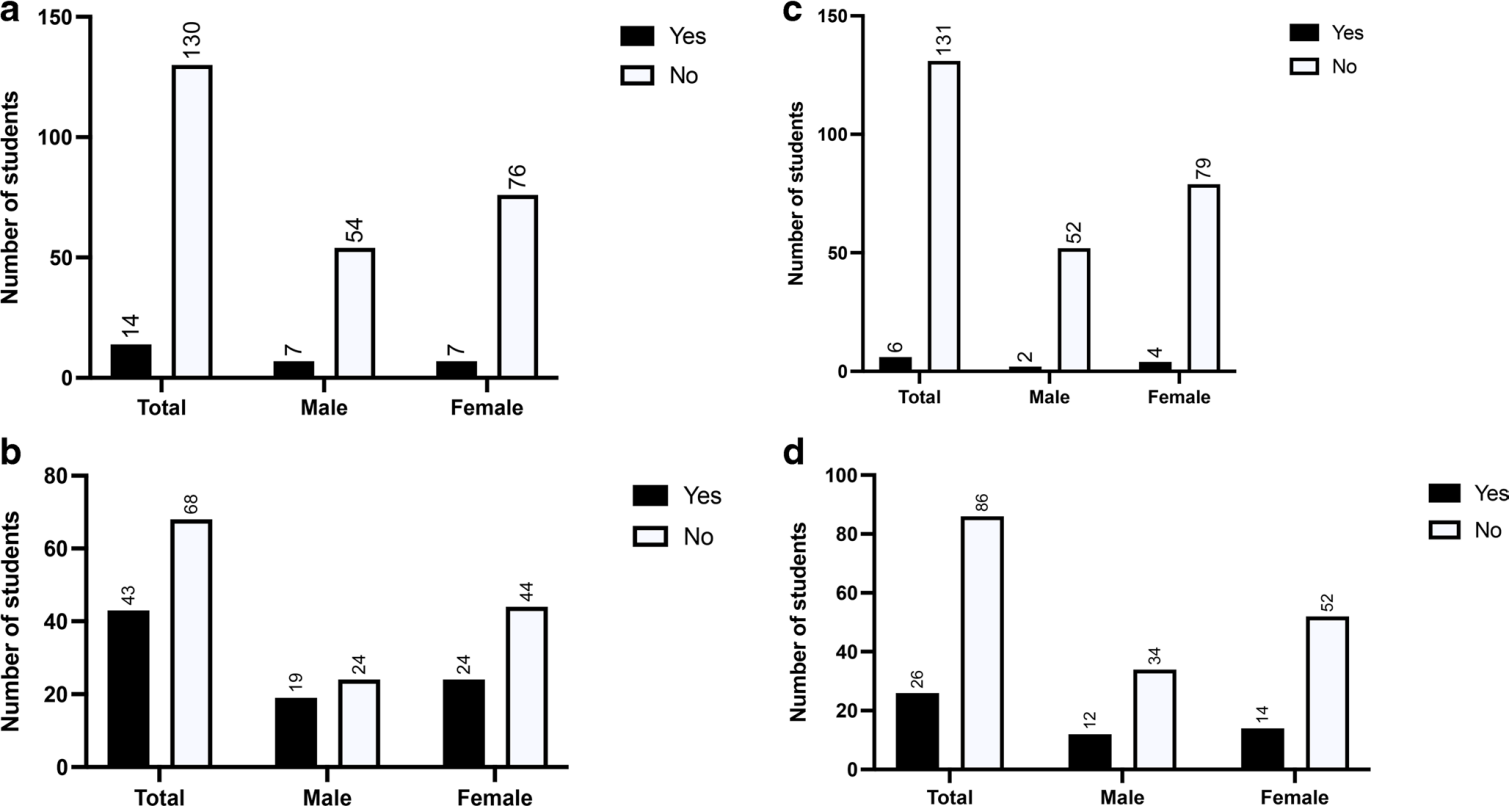
**a** Responses to the statement “I have already completed a clinical elective in a surgery department”. **b** Responses to the statement “I would like to do a surgical specialist training program after graduation”. **c** Responses to the statement “I have already completed a clinical elective in a department of anesthesiology”**d** Responses to the statement “I would like to do an anesthesia specialist training program after graduation”

Next, we asked the students how interested they are in starting a surgical or anesthesiologic specialist training after graduation; 61% (*n* = 68) of students said they did not want to become a surgeon (Fig. 3b) and 77% (*n* = 86) said that they did not want to become an anesthesiologist (Fig. 3d). We also asked if they have already completed a clinical elective in a department of surgery or anesthesiology, and 97% (*n* = 130) of students responded that they have not completed a clinical elective in a surgical department, while 96% (*n* = 131) responded that they have not completed a clinical elective in an anesthesiology department.

4. Career ambitions: What are the professional and academic career ambitions of medical students in their fourth year of study?

**Fig. 4:**
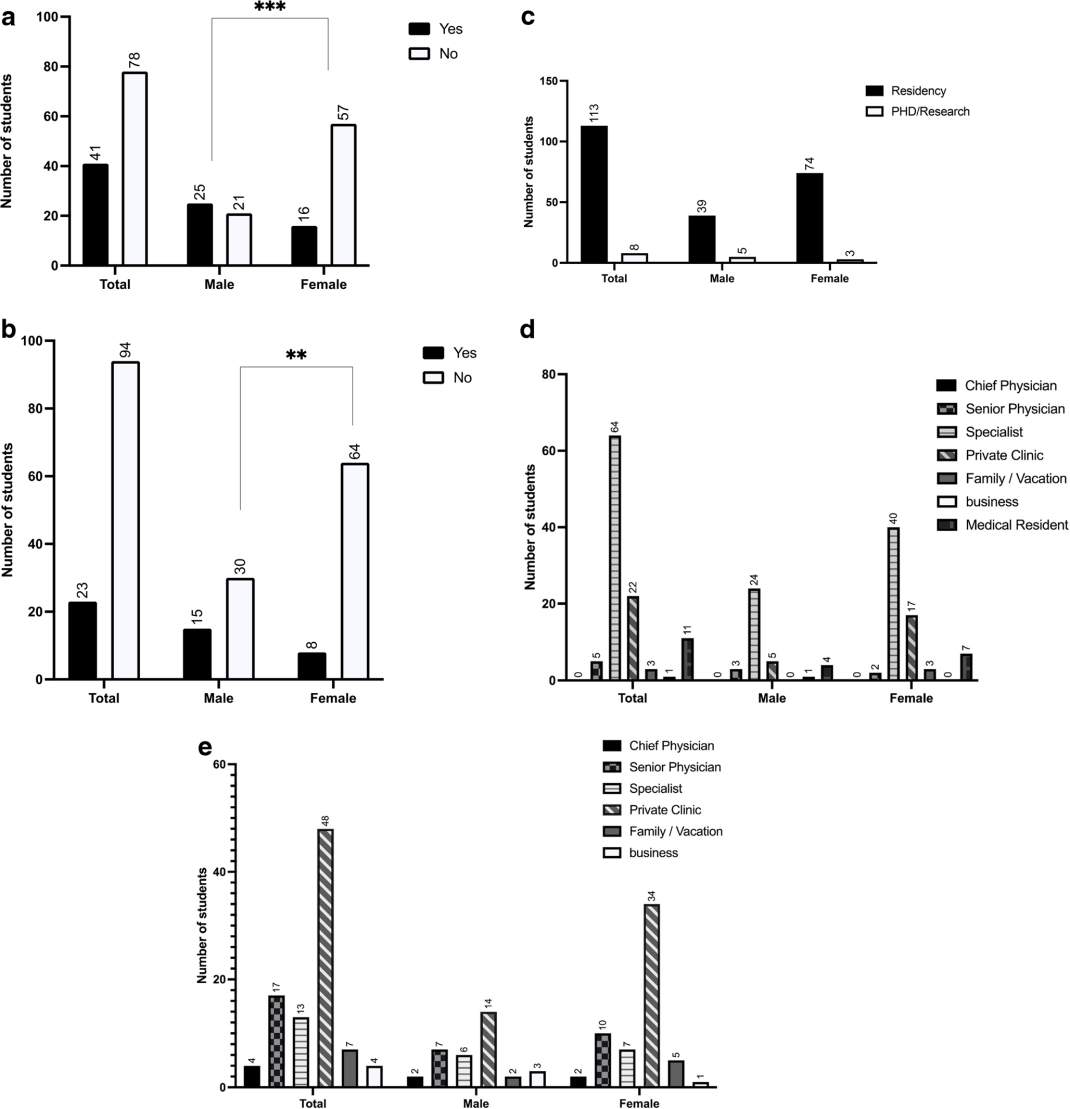
**a** Responses to the statement “I would like to get a habilitation.” *** P < 0.001. **b** Responses to the statement “My career goal is chief physician.” *** P < 0.001. **c** Responses to the question “Where do you see yourself in 5 years?”. **d** Responses to the question “Where do you see yourself in 10 years?”

Next, we asked the participants about their career goals over the next 20 years. Only a minority (34%, *n* = 41) of our study population said that they wanted to take an academic career path and get their habilitation, and significantly more male students than female students wanted to do this (54%, *n* = 25 vs. 22%, *n* = 16) (Fig. 4a). Furthermore, only 20% (*n* = 23) of participants said they wanted to be a chief physician, and significantly more males than females wanted this (*P* = 0.01) (Fig. 4b).

When asked where they see themselves in the next five years, 93% of students (*n* = 113) said they wanted to be in their residency while the remaining 7% (*n* = 8) said they wanted to be doing research or a PhD. When asked where they see themselves in ten years (*n* = 64), most participants saw themselves as specialists (38%, *n* = 24) or in a private practice (34%, *n* = 22). In 15–20 years, 52% (*n* = 93) of participants said they want to be working in a private practice (*n* = 48), and this option was twice as popular among female participants (*n* = 34) than among male participants (*n* = 14). The second most popular answer after private practice (albeit given by only 17 students) was being a senior physician.

5. Value concepts and wishes for future workplace: What values and wishes do medical students have for their future workplace?

**Fig. 5:**
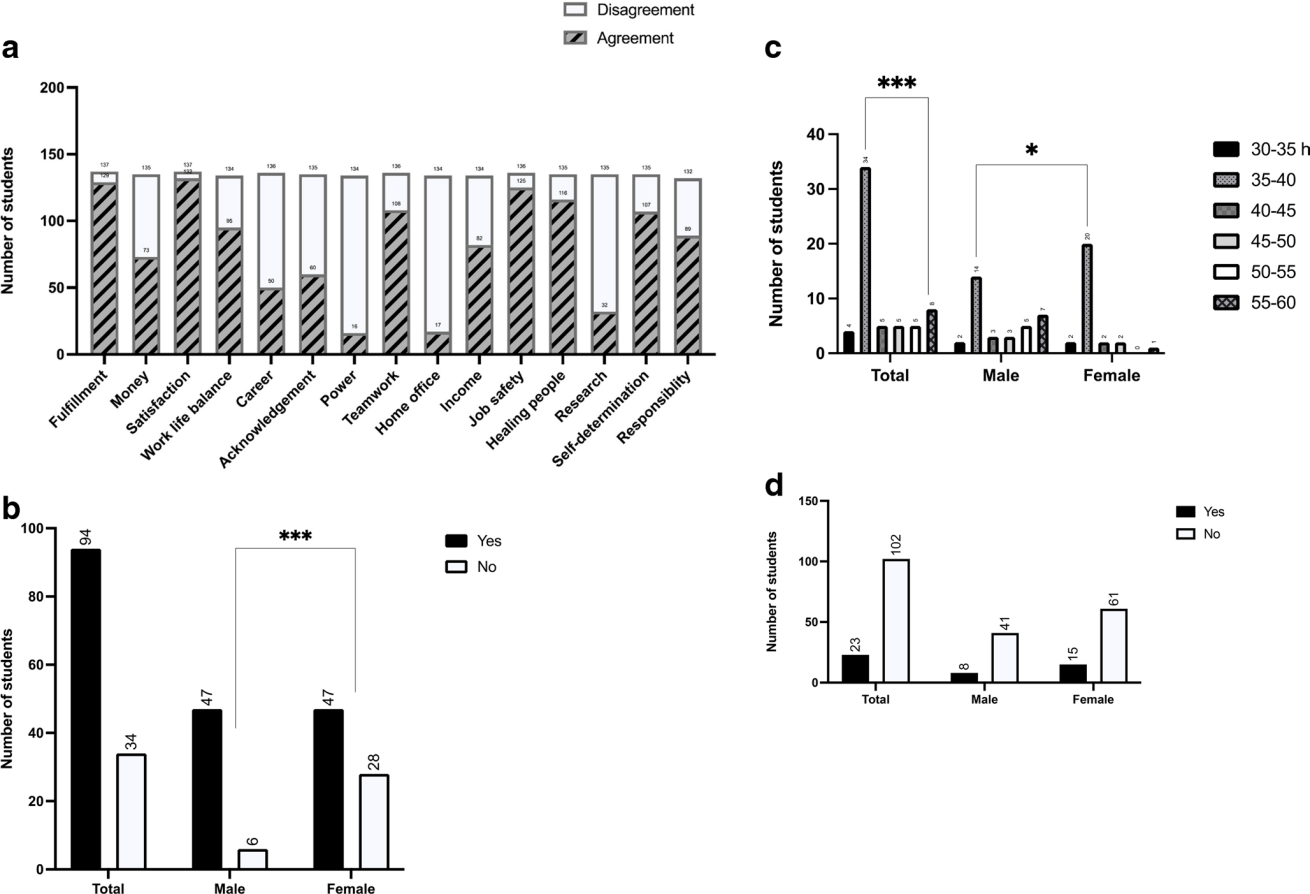

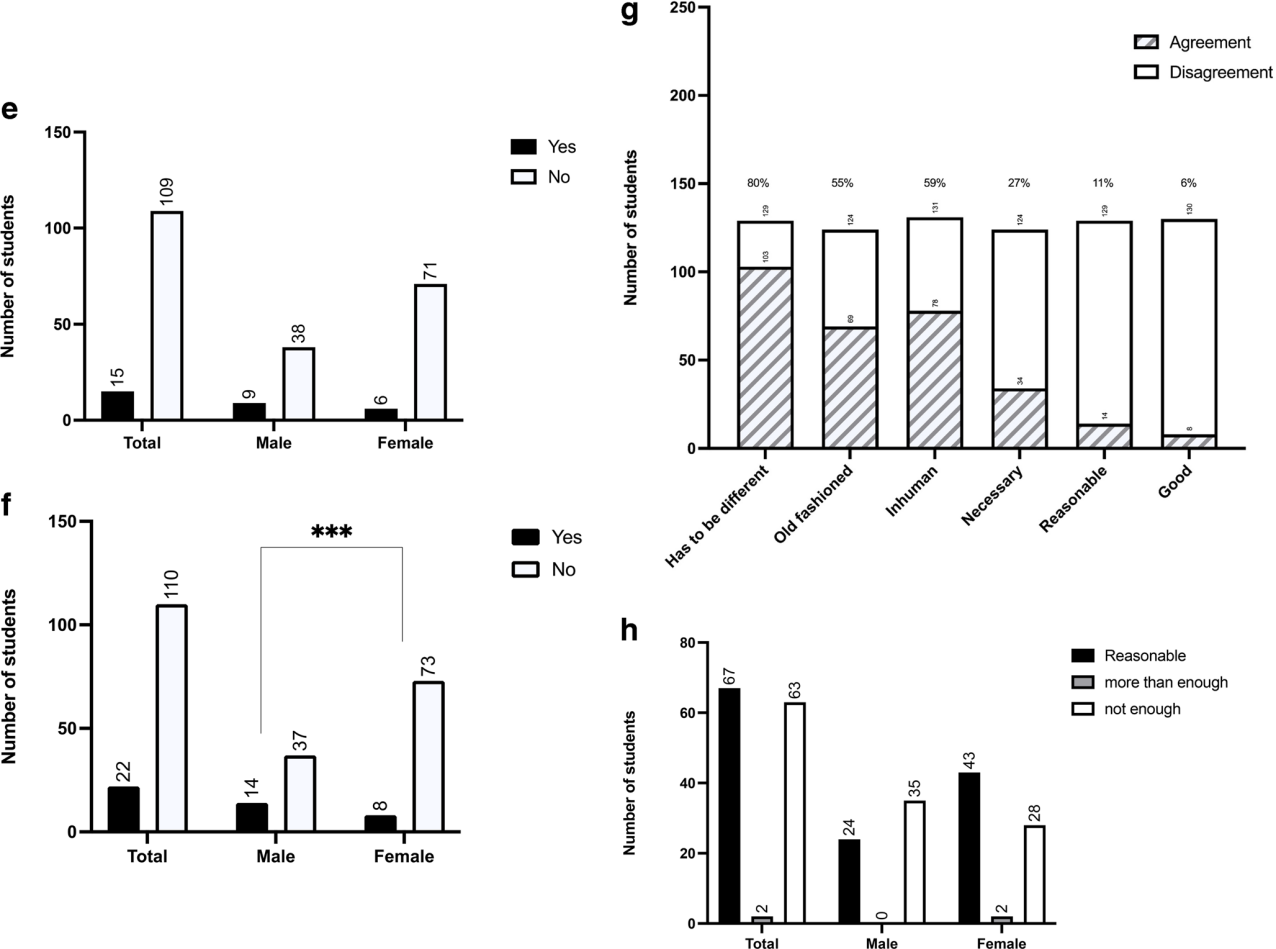
**a** Responses to the question “What is important for your job?” Disagreement = 1–3 , Agreement = 4–5. **b** Responses to the question “Do you want to work full-time after graduation?” *** P < 0.001. **c** Responses to the question “How many hours per week would you find appropriate as a full-time employee?”; (currently 42 hours according to the collective agreement). * P < 0.05, *** P < 0.001. **d** Responses to the statement “I want to work in a hospital lifelong”. **e** Responses to the statement “I would like to work without patients after graduation.” **f** Responses to the question “Can I imagine giving up my hobby/hobbies for my job?” *** P < 0.001. **g** Responses to the statement “24-hour shifts are…” where 1 = strongly disagree and 5 = strongly agree on the 5-point Likert scale (disagreement = 1–3, agreement = 4–5). **h** Responses to the statement “30 days holiday (for a full-time position) is appropriate/too much/too little for me”

Our next question was what values and wishes the medical students have for their future workplace. We gave the participants multiple options and more than one response was allowed. The most popular wishes for the future workplace were job satisfaction (96%), fulfillment (94%), job safety (92%), and healing people (86%). Options rated as less important were work-life balance (71%), money (54%), career options (37%) and research possibilities (24%) (Fig. 5a).

We also asked the participants if they want to work full-time after graduation. Significantly more female students said that they do not want to work full time (37%, *n* = 28) than the male students (11%; *n* = 6) (Fig. 5b). Furthermore, 67% (*n* = 84) of students said they want to work less than the statutory 42 h per week (Fig. 5c), and 81.6% (*n* = 102) shared the opinion that they do not want to work in a hospital lifelong (Fig. 5d). On the other hand, 88% (*n* = 109) of students stated that they did not want to work remotely from patients (Fig. 5e).

Another item in this question was whether the participants could imagine giving up their hobbies for their job? Overall, 83% (*n* = 110) of participants said they could not imagine giving up their hobbies for their future job. While only 10% (*n* = 8) of female students could imagine giving up their hobbies for their future career, 27% (*n* = 14) of male students were willing to do so (Fig. 5f). With regard to 24- hour shifts, 80% (*n* = 103) shared the opinion that 24-hour shifts are obsolete, 59% (*n* = 78) added that these long shifts are inhumane, and 55% (*n* = 69) said that working 24 h non-stop is old fashioned. Only 11% (*n* = 14) of participants said they thought 24-hour shifts are reasonable and only 6% (*n* = 8) shared the opinion that these shifts are good, while 27% (*n* = 34) acknowledged that they think these long shifts are necessary (Fig. 5g). We also asked the participants if 30 days of vacation a year is enough for them (Fig. 5h). Half of the participants said this is appropriate (*n* = 67), while 48% (*n* = 63) believe that 30 days of vacation per year is not sufficient and that 40–42 days would be more suitable (Fig. 5h).

## Discussion

In this study, we examined the generation gap between Gen Z medical students and their medical teachers. We found that Gen Z medical students show less career ambition, prefer online learning tools, and want more breaks and longer holidays than earlier generations did. These needs and wishes of Gen Z medical students have been shaped by their digital upbringing, which has influenced their expectations regarding communication and collaboration. It has been shown that this generation tends to favor flexible working hours, remote working, and open communication [20]. Furthermore, they are keen to develop their skills and engage in lifelong learning, and expect their employers to provide this [21]. They also place considerable importance on diversity, inclusion, and social responsibility [22].

In our survey, Gen Z medical students most often gave social reasons for studying medicine, namely job satisfaction, fulfillment, job safety, and healing people. Interestingly, earning money or building a career played a less important role in the decision to study medicine. When asked about their career intentions, nearly all participants had the same five-year intention—to pursue their specialist training. When asked about their ten-year career intentions, it became clear that private aspects such as taking vacations and having a family are far more important to this generation. Astonishingly, only a few participants were interested in becoming a chief physician. Many of our participants imagined themselves as medical practitioner instead. We were also surprised to see that almost none of the students wanted to pursue surgical or anesthesia training, possibly because of the less flexible working conditions. There was also very little interest in research; only a very small number of students wanted to work scientifically or were interested in a research fellowship. These findings are in agreement with the findings of others, that Gen Z individuals want to work independently and at their own speed [4, 23, 24].

Most of our participants wanted to work with patients, but did not want to be restricted to lifelong work in a hospital. This desire to work with patients contrasts the findings of others. For example, Holzer et al., reported that high school students planning to study medicine were interested in quitting patient care later on [15, 25], although this study population was younger than ours and may change their opinions with time. In addition, Streit et al. reported that more physicians have left patient care over the last decades [25]. Moreover, it has be discussed if they really have lower career ambitions or if it is rather a changed understanding of career.

Surprisingly, although almost none of the students has opted for a clinical traineeship in visceral surgery or anesthesia and therefore do not know the subject area at all, most of the students stated that they do not want to do specialist training in one of the two fields maybe due to less flexible working conditions.

Our questionnaire indirectly addressed the mental health of students during medical studies. For example, we found that students need a break after working for 3.2 h on average, and that they do not want to work 24-hour shifts. In addition, most students said they did not want to work full time, and over half thought that 30 days of vacation per year is not enough. These findings reflect an important need for more personal time for physical and mental regeneration among Gen Z medical students. Earlier studies have shown that 82% of medical students showed signs of psychological distress, and nearly half experienced burnout. Alarmingly, it has also been reported that 11.2% of medical students admitted thoughts of suicide [13, 26, 27]. However, this tendency towards psychological disorders is not limited to medical students but seems to affect the Gen Z population as a whole, indicating that this generation is more prone to psychological distress than earlier generations were. To address this problem in medicine, students could be offered a personal mentor to help them strengthen their resiliency [13, 28–38].

The needs and wishes of Gen Z students we have uncovered constitute challenges for future employers. Most chief physicians and senior medical staff in hospitals are Gen X, and have experienced a traditional education and medical career. This means there are often differences in communication, leadership, and collaboration between Gen X and Gen Z (Jenei et al., 2024), which can lead to challenges in the workplace. Our results have indicated that feedback and good communication are important for medical students in their future workplace. The Gen Z employee has been socialized digitally, so has different expectations of collaboration. Our findings show that they prefer flexible and agile working forms, whereas the Gen X employee often prefers more traditional forms of working [4]. These diversities present important challenges in the employer-employee relationship.

Based on our results, we have some suggestions for addressing these challenges presented by the generation gap. First, medical students could be assigned a personal clinical mentor who can support their mental wellbeing and give important feedback. Second, the social expectations of future employees need to be met, for example through flexible working time models and onboarding after a period of leave (such as parental leave or research leave). Finally, we could offer an early mentoring program in medicine that includes a research track to support and guide those with academic career ambitions.

### Limitations

There are some limitations to our study. One limitation is that the questionnaire was only completed once by each medical student during their surgical semester, so we did not investigate how students’ needs and wishes change over time. Another limitation is that the study was conducted at a single medical faculty, which limits the generalizability of the findings to other institutions or countries. A third limitation is that we did not perform a formal sample size or power calculation and simply invited fourth-year medical students to participate, so our sample size may be too small to detect a true effect. Future multicenter studies with larger sample sizes are needed to enhance validation, reliability, and generalizability and to ensure a representative sample. These studies should evaluate the needs and wishes of medical students multiple times and over the entire course of medical studies to determine how needs and wishes change.

Another limitation is that the questionnaire was filled out only by Gen Z medical students, so we cannot compare the opinions of the students with those of working Gen X and Gen Y physicians, lecturers, residents, and consultants. These generation comparisons should be made in future studies to emphasize differences and to identify collaboration problems in the workplace. A further limitation is that we did not randomize the participants and that the questionnaire was self-designed, which could have resulted in bias. We reduced the chance of bias by making the questionnaires anonymous and by phrasing the questions in a neutral way. Given the descriptive and exploratory nature of the study, the potential impact of common method bias on the findings is likely limited. In addition, because multiple statistical comparisons were performed without adjustment, the possibility of type I error cannot be excluded. Statistical comparisons should therefore be interpreted as descriptive and hypothesis-generating rather than confirmatory. Furthermore, the dichotomization of Likert-scale responses facilitated interpretation but may have resulted in a loss of information compared with analyses using the full ordinal scale.

Another limitation of our study is that we categorized our participants as Gen Z based on their year of birth, which some argue is an arbitrary categorization that is not scientifically sound. It is important to understand that an individual’s identity and behavior is not crystallized or ratified by the particular time they were born, rather they evolve throughout life based on intersections between age, life stage, and social context. It is this that creates varying attitudes, values, and behaviors, not an individual’s generation. Furthermore, the attitudes we observed in our participants (such as the desire for part-time work, rejection of 24-hour shifts, and lack of interest in research) could be explained by the structural conditions of current medical training and the working environment in general rather than generational preferences. Although generational research can help us understand and manage the diverse workforce, it also oversimplifies the complexities inherent to both individuals and the environments in which they live and work [39]. That said, it is important to address the changing mindset and goals of Gen Z medical students during their studies and residency. At least may personal opinios about generational concepts have an impact in surveys through identification and reactions to labels or frames. This can shift atttitudes and response tendencies. Those who perceive generationalism as “bad science” or a stereotype machine tend to react with contradiction, downplaying, or distancing themselves—regardless of the actual content [40]. One american study found that the majority of Americans actually identify with the “correct” labels for their generation, as defined by the Pew Research Center based on their year of birth [41]. However, an important finding of this study is the remarkable extent of intragenerational differences in identification with the group label. Within each generational group, there is a predictable pattern, with the likelihood of identification increasing from the early years, peaking in the middle of the cohort, and then gradually declining again in later years. Among all generational labels, the baby boomer cohort showed the highest level of identification. Although the peak was lower for each subsequent generation, the other generations showed a similar trend. It is noteworthy that these trends remained consistent even after taking into account the demographic composition of the cohorts, such as ethnicity, education, income, gender, and region. Embracing Generational Labels: An Analysis of Self-Identification and Political Partisanship [42].

## Conclusion

This study highlights a changing mindset in Gen Z medical students on what is important to them in their life and career. This generational shift is not merely a challenge to “modernize” teaching but also an invitation to redesign curricula, learning environments, and support systems so that they consider the students’ mental health status and are humane, digitally competent, and sustainable for future generations.

Gen Z has new expectations of its employers. For the healthcare sector to succeed, employers and mentees should adapt to the needs of this younger generation. This can be achieved by aligning with the digital culture, promoting flexibility and autonomy, providing purpose and meaning, facilitating continued learning and development, and supporting a healthy work-life balance [10, 36]. For example, supervisors and employers could incorporate digital tools into the learning program, provide a more flexible working time table, offer onboarding help after parental leave, and allow employees to work from home where possible. We also believe that a mentorship program could increase enthusiasm about career progression by providing both clinical and research support.

## Data Availability

All data supporting the findings of this study are available within the paper and its Supplementary Information.
